# Effects of a Dynamic Warm-Up, Static Stretching or Static Stretching with Tendon Vibration on Vertical Jump Performance and EMG Responses

**DOI:** 10.2478/hukin-2013-0067

**Published:** 2013-12-31

**Authors:** Bulent Yapicioglu, Muzaffer Colakoglu, Zafer Colakoglu, Halil Gulluoglu, Fikret Bademkiran, Ozgur Ozkaya

**Affiliations:** 1 Ege University, School of Physical Education and Sports, Department of Coaching Education, Turkey.; 2 Ege University, Faculty of Medicine, Dept. of Neurology, Division of Clinical Neurology, Izmir, Turkey.; 3 18 Mart University, Faculty of Medicine, Dept. of Neurology, Division of Clinical Neurology, Canakkale, Turkey.

**Keywords:** Exercise, reflex, silent period, surface electromyography

## Abstract

The purpose of this study was to investigate the short-term effects of static stretching, with vibration given directly over Achilles tendon, on electro-myographic (EMG) responses and vertical jump (VJ) performances. Fifteen male, college athletes voluntarily participated in this study (n=15; age: 22±4 years old; body height: 181±10 cm; body mass: 74±11 kg). All stages were completed within 90 minutes for each participant. Tendon vibration bouts lasted 30 seconds at 50 Hz for each volunteer. EMG analysis for peripheral silent period, H-reflex, H-reflex threshold, T-reflex and H/M ratio were completed for each experimental phases. EMG data were obtained from the soleus muscle in response to electro stimulation on the popliteal post tibial nerve. As expected, the dynamic warm-up (DW) increased VJ performances (p=0.004). Increased VJ performances after the DW were not statistically substantiated by the EMG findings. In addition, EMG results did not indicate that either static stretching (SS) or tendon vibration combined with static stretching (TVSS) had any detrimental or facilitation effect on vertical jump performances. In conclusion, using TVSS does not seem to facilitate warm-up effects before explosive performance.

## Introduction

The pre-activity warm-up is a widely accepted exercise to help prepare athletes, both physically and mentally, for maximum performance. Static stretching (SS) is a technique that is often incorporated into warm-up routines because it is known to increase the range of movement (Cross and Worrell, 1999), which is beneficial to athletes who require higher levels of flexibility (Shellock and Prentice, 1985). However, some studies showed that using static stretching before explosive exercise may deteriorate power production (Cramer et al., 2007), vertical jump height (Wallmann et al., 2005) and sprinting performance (Fletcher and Anness, 2007), whereas dynamic stretching may enhance power development and VJ performance (Kokkonen et al., 1998). Therefore, in other studies, it is suggested that static stretching included in pre-competition warm-up programs should be replaced by dynamic stretching because SS might diminish muscular power production (Young and Behm, 2003; Wallmann et al., 2005). This may be based on stretching-induced force deficits due to mechanical factors such as decreases in muscle tension and neural inhibition resulted from tendon reflex (Herda et al., 2009).

Another popular application aimed at enhancing performance is vibration training. Recent research shows that vibration training may improve neuro-muscular performance (Bosco et al., 1999; Cardinale and Bosco, 2003; Cardinale and Lim, 2003; Cardinale and Wakeling, 2005). This improvement may result from recruitment of previously inactive motor units (Mischi and Cardinale, 2009), enhanced motor excitability (Cardinale and Bosco, 2003; Delecluse et al., 2003), increased muscle temperature and blood flow (Bosco et al., 1999) as well as facilitating neural functions resulting from tonic vibration reflex (Lapole and Perot, 2010). Tonic vibration is known to attenuate inhibitory effects of tendon reflex stem from SS. In order to address the controversy of using SS in pre-competition warm-up protocols, we added vibration with SS to observe if vibration might diminish the negative effects of SS. Therefore, we aimed to investigate the potential effects of tendon vibration on effects of SS on vertical jump performance.

## Material and Methods

### Participants

Physically active and healthy male college students (n=15; age: 22±4 years old; body height: 181±10 cm; body mass: 74±11 kg) with vertical jump performance above 40 cm were voluntarily recruited to the study. Sports participation backgrounds of volunteers were 8.7±2.3 years. Soccer players (n=10) and track and field athletes (n=5) participated in the study. For the time of the experiment all participants attended two to six training sessions per week in their particular sports disciplines. No injuries resulted and all volunteers were able to complete the study. The study protocol was approved by the local ethics committee.

### Experimental Design

**Figure 1:**
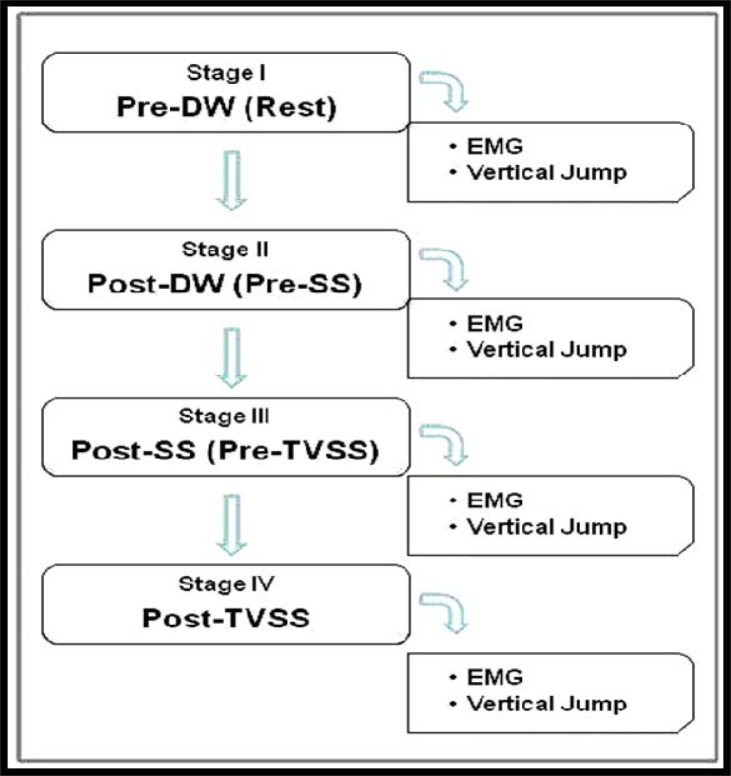
***Flow chart of study procedures.***

EMG and vertical jump data were obtained from the following succeeding stages: *I)* pre-DW; *II)* post-DW / pre-SS; *III)* post-SS / pre-TVSS and *IV)* post-TVSS. Vertical jump tests were realized immediately after EMG analyses. Although a traditional routine pre-competition warm-up includes a dynamic warm-up as well as static stretching protocols, this process directly effects the real competition performance. Therefore, it was not deemed suitable to give rest between any study stages to simulate the real competition environments and to observe acute effects of applied vibration. All stages were completed in 90 minutes for each participant. The flow chart of the study was shown in Figure 1.

### Procedures

#### Dynamic warm-up

The dynamic warm-up consisted of three minutes jogging and dynamic stretching with no static holds. All subjects walked 15 m between exercise periods (Table 1).

#### Static stretching

Static stretching exercises for the Achilles tendon consisted of eight different 30 s stretching exercises totaling four minutes. All subjects were pushed by a senior assistant to reach “subjects’ pain threshold” so muscle-tendon systems were stretched to their limits without injury. Once this point was reached, the stretch was held for 30 s and then repeated for the other leg.

#### Tendon vibration

While in the standing position, the participants leaned against the wall without losing the straight line between their head, neck, spine, pelvis, rear leg and ankle. They kept their rear foot down and parallel to their hips, bended their arms, and shifted their weight toward the wall. While they were stretching their Achilles tendons, tendon vibration was applied by means of a direct vibration device (TVR, HV-12D, Heiwa Electronic Industrial Co. Ltd., Denshi, Japan) using an amplitude of 1.0 to 2.0 mm and frequency of 50 Hz (Lapole and Perot, 2010), for 30 seconds.

#### EMG analysis

H-reflex parameters (H-reflex threshold, H-M latency and H/M amplitude ratios), amplitude alterations of T-reflex and peripheral silent period responses to popliteal post tibial nerve electro-stimulation were recorded (Nicolet Viking IV, Nicolet Corp., Madison, Wisconsin, USA) by single differential pre-gelled Ag/AgCl surface electrodes. Skin surface was carefully prepared before the electrode placement by shaving, removing the dead layer of the skin along with its protective oil, and cleaning the surface with alcohol to lowered electrode impedance ≤10kΩ (Clarys and Cabri, 1993). While the participants were lying prone, a bipolary stimulation electrode (Medtronic Electrodes, 9 mm × 6 mm) was placed at left popliteal and a recording (Viasys Electrodes, 20mm × 7mm) electrode was placed at midline on the left soleus muscle belly. The ground electrode (Stainless Disc Ground Electrodes, 40 mm size) was placed between the popliteal fossa and soleus muscle belly. Possible cable motion artifacts and power line interferences were minimized by using an adhesive bandage throughout testing.

Electrical stimulation was applied by a constant current stimulator (Nicolet Biomedical, A, IES 405-2, Isolated Electrical Stimulator, USA) delivered by single square electrical pulses (1 msec, max. intensity 400V) to the posterior tibial nerve. Before the M wave, maximum amplitude was elicited with maximum stimulus, stimulus amplitude declines were determined manually, and the point of first appearance and missing H-reflexes were established. Once the H-reflex was elicited, the maximum amplitude (peak to peak) of the M wave (Mmax) was determined. All stimulus were given non-recurrent. Once the electrode position and stimulation intensities had been determined, the electrodes were secured with a strap and a bandage to minimize the risk of displacement throughout testing.

**Figure 2:**
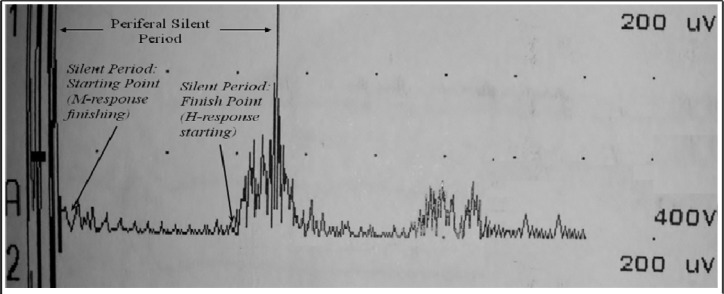
***Starting and final points of peripheral silent period (Trace is rectified)***

First negative deflection was accepted at the point of potential starting. After the initial H-reflex was attained, electrical intensities were increased randomly. H response was reached first, then, by increasing electrical stimulation, M responses were reached. The first electrical stimulation resulting in an H-reflex was accepted as the threshold. The values of max H and max M were estimated. The difference between M and H potential starting latency was accepted as the HM latency difference. H-reflex frequency values were between 2 Hz-5 kHz and sweep time was applied at 50 msec. As a stimulator, the Disa-Type 15B01 model reflex hammer was used. The frequency values were between 2 Hz–10 kHz and the sweep time was applied at 100 msec. The measurements were taken by applying surface electrodes to the popliteal posterior tibial nerve of the soleus where the maximal M response was found. After stimulation, the area where motor activities ceased was accepted as the silent period starting point. After the silent period, the point where the motor unit activities started was accepted as the final point (Figure 2). Four measurements were taken randomly from each subject and the shortest silent period was used in analyses. The frequencies were between 2–10 kHz. The sweep time was 500 msec.

#### Vertical jump measurements

In the VJ procedure, a New Test 2000 contact mat system (New Test Oy., Oulu, Finland) was used to measure jump height (cm). Subjects were instructed to begin in a standing position and the jump technique was demonstrated to them. A modified counter movement jump, with hands positioned on the hips, was used to measure VJ in order to exclude the inertial effects of arm movements. The best VJ performance was recorded after three trials for each participant.

### Statistical analyses

Results were analyzed using the SPSS version 17.00 (SPSS Inc., Chicago, USA). The Shapiro-Wilk test was used to conduct ANOVA testing on the data. Bonferroni pair-wise comparisons were used for post-hoc analysis to determine differences between groups. Pearson correlation coefficients (r) were used to determine the linear relationship between tested variables. Findings with a p<0.05 were considered statistically significant.

## Results

**Figure 3:**
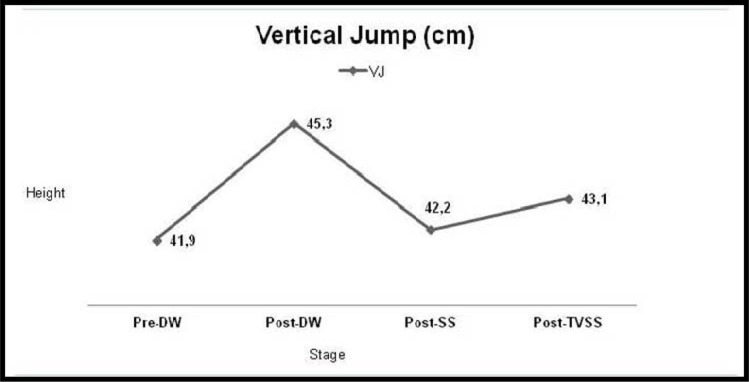
***Tested vertical jump findings during succeeding stages such as pre-DW, pos-DW, SS and TVSS***

**Figure 4:**
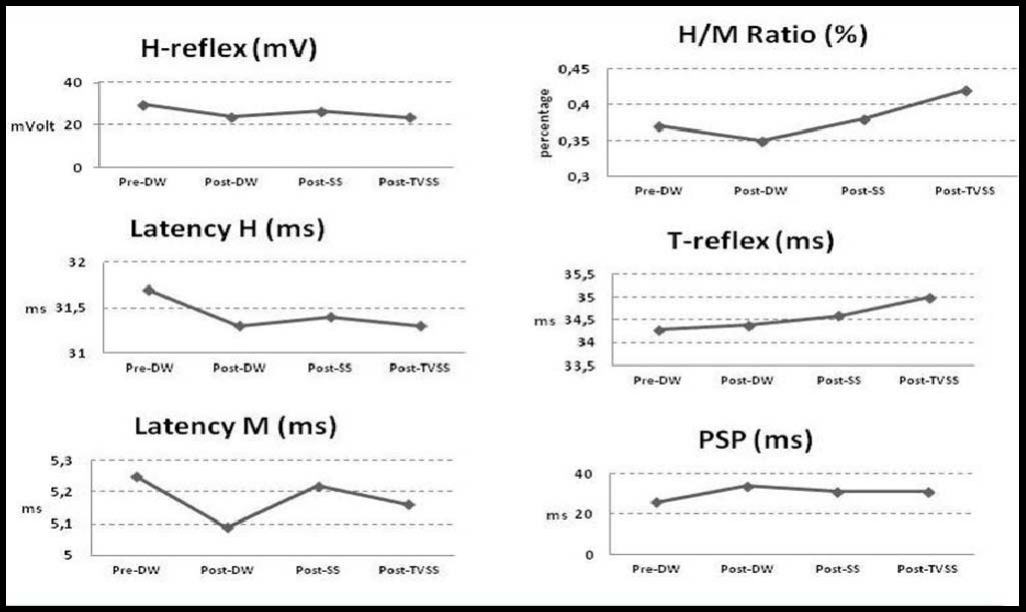
***Tested EMG parameters such as Hoffmann reflex (H-reflex), H/M ratio, tendon reflex (T-reflex) and peripheral silent period (PSP)***

Vertical jump was significantly higher after the DW than at the baseline (42±6 vs. 45±6 cm, respectively; p<0.05) (Table 2, Figure 3). Post-SS and TVSS VJ heights were not significantly higher than the baseline or post-DW values (p>0.05) (Figure 4). None of the EMG parameters was significantly different from each other at any time: pre-DW, post-DW, post-SS or post-TVSS (p>0.05).

## Discussion

The purpose of this study was to investigate short term performance outcomes and neurological effects of static stretching, the dynamic warm-up (DW) and tendon vibration combined with static stretching (TVSS). The most interesting result was a significant increase of VJ performance following the DW (p=0.004). Improvements in VJ performance may be related to the optimal muscle and body core temperature, enhancement of motor excitability, improvement of kinesthetic awareness, maximizing increments of active ranges of motion, development of fundamental movement skills (Young and Elliott, 2001; Faigenbaum et al., 2006) and nervous system stimulation (McMillian et al., 2006; Hough et al., 2009).

In the next stage, after the dynamic warm up, static stretching (SS) was performed by each subject. After the execution of only SS, there was no significant change in VJ performance (p>0,05). This is similar to the findings of other researchers who did not find negative influences of SS on power (Power et al., 2004), strength (Kokkonen et al., 1998; Fowles et al., 2000) nor VJ performance (Young and Behm, 2003; Wallmann et al., 2005). Effects of SS on muscular force production capabilities are still a controversial topic. Many studies indicate that SS may decrease muscle strength (Fowles et al., 2000) and explosive power (Young and Elliott, 2001; Bradley et al., 2007) but other research does not support a negative influence of SS applied before exercise (Power et al., 2004; Manoel et al., 2008).

We expected to find a negative effect of SS on VJ performance because of a possible inhibitory effect on the Golgi tendon organ. However, our four minute application of SS did not result in a negative effect on VJ. The lack of an inhibitory effect might possibly be explained by differences in the pain threshold levels of subjects which may have resulted in inadequate execution of the stretching exercises. Subjects were asked to perform the stretch movement until they experienced pain and then to hold the stretch for 30 seconds in this position. Since pain thresholds and flexibility levels differ from person to person, variations in the execution of SS may have prevented tendon inhibition.

In the final stage of this study, the combined effects of direct vibration and SS execution were investigated. Some researchers have found a negative effect of combined static stretching and vibration (Herda et al., 2009), but the combined vibration with SS application did not yield negative effects in the present study. Vibration (50 Hz, 1.0–2.0 mm displacement) was directly applied to the Achilles tendon during the 30 second SS and no significant change was found in the VJ performance after this application (p>0,05). Kinser et al. (2008) investigated the effects of simultaneous vibration and static stretching on flexibility and explosive power in 22 young female competitive gymnasts. They applied direct vibration (30Hz, 2mm displacement) at four sites combined with 10 second SS and allowed 5 seconds of rest between sets. They showed that acute vibration–stretching resulted in significantly different increment levels in flexibility (p<0.05) compared with vibration-only treatments. Explosive power variables such as peak force, rate of force development, jump-height, flight time and instantaneous forces over the jump were not statistically different in pre/post jump performance in either the counter movement jump or squat jump (p>0.05). There was no significant change in power parameters of jump performances after the application of 30 Hz-vibration frequency (10sec × 4 sets) combined with SS (Kinser et al., 2008). We expected to observe a positive effect on jump performance and EMG parameters after a one set application of 50 Hz-vibration lasting 30 seconds, but no such effect was found in jump performance or EMG parameters (p>0.05).

Herda et al. (2009) examined the acute effects of passive stretching vs. prolonged vibration on voluntary peak torque, peak twitch torque, passive range of motion, and surface EMG amplitudes of the medial gastrocnemius and soleus muscles during isometric maximal voluntary contractions of the plantar flexors in 15 healthy moderately trained men. Although there are some differences in methodological considerations based on a total 20-min duration of passive stretching (135-s × 9 reps), a duration of 20-min 70Hz Achilles tendon vibration, according to results of their study, maximal isometric voluntary contractions were not affected by passive stretching or tendon vibration in all control, passive stretching and tendon vibration groups. Indeed, our results showed that explosive power production was not affected by acute static stretching or vibration applications. However, we decided on different duration of static stretching treatments, different duration and frequency of vibration applications, etc. These results may not be sufficient to explain underlying mechanisms of stretching induced force deficit or the effects of tendon vibration, it seems that there are more studies needed with different treatment durations and frequencies to explain this phenomenon (Herda et al., 2009).

Improvement in physical performance after vibration has been primarily attributed to neural factors, such as increased motor unit synchronization (Martin and Park, 1997), stretch reflex potentiation (Jordan et al., 2005), increased synergist muscle activity and increased inhibition of the antagonist muscle (Bosco et al., 2000; Cardinale and Lim, 2003). In this study we used a tendon vibration device instead of vibration dumbbells or platforms since it may be more effective to facilitate tonic vibration reflex neural functions due to the proximity of vibration source to targeted muscle (Luo et al., 2005; Lapole and Perot, 2010). In addition, smaller and portable tendon vibration devices may be more preferable than much heavier and larger whole body vibration platforms in performance settings. Although we presumed that TVSS applied subsequently after SS might have compensated for the negative effects of SS on VJ and EMG responses, our results showed that these parameters remained unaffected after either SS or TVSS. Arithmetical variations in analyzed parameters indicate that tendon vibration may help to recover from inhibitory effects of SS. It seems that neither SS nor tendon vibration combined with SS had an effect on explosive power and EMG parameters of moderately trained volunteers.

In conclusion, results from the present study could not provide definitive findings in solving the controversy. In future studies, effects of static stretching and tendon vibration on selected performance and/or physiological parameters should be investigated in both moderately and highly trained subjects forming larger groups. In addition, studies aimed at determining the most efficient combination of frequency, duration and volume of vibration application combined with static stretching could provide useful knowledge in this research field.

## Figures and Tables

**Table 1:** ***Dynamic stretching procedure***

Warm-up	**Description**	**S**	**T / D**
	Jogging	1	3 min
Elbow to opposite knee	Subjects stood with stick in one hand, then lifted one knee towards the opposite shoulder while bringing that elbow towards the knee and touched it.	1	8 reps per leg
Skips	Subjects performed double leg skips repeatedly with a linear forward movement.	2	15m
High Knee Skip Bounds	Subjects stood with feet slightly wider than shoulder width and kept their hands at waist level. They performed a quick shuffle of feet until the call for high knees (run in place).	2	15m
Toe Touch and Walk	Subjects extended one leg straight out in front of the body and touched their knee/toes and did the same with the other leg during the next step.	2	15m
Heel Kicks	Subjects rapidly kicked heels toward buttocks while moving forward.	2	15m
Fast Butt Kicks	Subjects slightly kicked their buttocks with their heels. Butt kicks are performed with proper sprint arm action.	2	15m
Lateral Shuffles	Subjects took a semi-squat position, then performed a lateral shuffle with a long first step followed by a second quick step.	2	15m
Carioca	Subjects performed high knee carioca motions. The rotation occurred below the hip while the torso remained relatively perpendicular to the direction of acceleration.	2	15m
Back Pedal	Subjects back pedaled with small quick steps maintaining a tall braced core, a flat back and proper arm movements.	2	15m

R:Repetation, T: Time, D: Distance.

**Table 2:** ***Variations amongst VJ and EMG parameters***

Parameters	Pre-DW	Post-DW/Pre-SS	Post-SS/Pre-TVSS	Post-TVSS
VJ	41.9±5.7^a^	45.3±6.2^a^	42.2±5.5	43.1±4.7
H-Threshold (mV)	29.5±23.2	23.7±12.4	26.5±12.3	23.6±9.6
Latency H (ms)	31.7±2.2	31.3±2.3	31.4±2.6	31.3±2.3
Latency M (ms)	5.25±0.71	5.09±0.70	5.22±0.78	5.16±0.62
H/M Ratio (%)	0.37±0.21	0.35±0.25	0.38±0.22	0.42±0.23
T-Reflex Latency (ms)	34.3±3.1	34.4±3.0	34.6±2.8	35.0±2.8
PSP (ms)	25.9±16.2	33.9±18.1	31.1±16.7	31.3±18.3

H-reflex: Hoffmann reflex; Post-DW/Pre-SS: After dynamic warm-up/before static stretching; Post-SS/Pre-TVSS: After static stretching/before tendon vibration with static stretching; Post-TVSS: After tendon vibration with static stretching; Pre-DW: Before dynamic warm-up; PSP: Peripheral silent period; T-reflex: Tendon reflex; VJ: Vertical jump.
